# Activity of *Porophyllum ruderale* leaf extract and 670-nm InGaP laser during burns repair in rats

**DOI:** 10.1186/s12906-015-0805-2

**Published:** 2015-08-13

**Authors:** Ana Cristina Justino Jácomo, Karina de Andrade Velozo, Raquel Gabilan Lotti, Lia Mara Grosso Neves, Fernanda Oliveira de Gaspari de Gaspi, Marcelo A. Marreto Esquisatto, Maria Esméria Corezola do Amaral, Fernanda A. Sampaio Mendonça, Gláucia Maria Tech dos Santos

**Affiliations:** Graduate Program of Biomedical Sciences, Hermínio Ometto University Center, UNIARARAS, Av. Maximiliano Baruto, 500, CEP 13607-339 Araras, SP Brazil

**Keywords:** *Porophyllum ruderale*, Laser, Burns, Tissue repair, Rats

## Abstract

**Background:**

In this study, we investigated the effects of an extract of the leaves of *Porophyllum ruderale* and laser irradiation on the healing of burns.

**Methods:**

Seventy-two rats were divided in four groups: untreated controls, treated with laser irradiation, treated with *P. ruderale* and treated with both *P. ruderale* and laser irradiation. Burns were produced with a metal plate on the backs of the animals. Wound samples were collected for structural and morphometric analyses and to quantify the expression of TGF-β1 and VEGF.

**Results:**

Laser irradiation increased the number of fibroblasts, collagen fibers and newly formed vessels and decreased the number of granulocytes at the site of the wounds. Densitometric analysis revealed a significant increase in the expression of TGFβ-1 in the wounds treated with laser irradiation and with the *P. ruderale* extract at the beginning of the healing process and a decreased during the experimental period. The expression of VEGF was highlighted in the lesions irradiated with laser alone.

**Conclusion:**

Inspite of not showing a beneficial effect on the laser combination with the *P. ruderale* extract, when the laser was used separately, a positive effects to enhance the healing of second-degree burns was promoted. *P. ruderale* was effective in decreasing the granulocytes during the repair process indicating a possible anti-inflammatory action of this extract of native flora, widely used in folk medicine, but little studied experimentally.

## Background

The healing of burn wounds has long been a research focus because of the problems associated with this type of injury, such as time of healing, infections, morbidity and mortality. In second-degree burns, the epidermis and superficial dermis are the main layers affected. In addition to destruction of the epithelial barrier, the presence of degraded proteins and devitalized tissue provides an excellent environment for the growth and proliferation of microorganisms [1] which are a serious public health problem.

All phases of repair are mediated by molecular signals, including specific growth factors, which stimulate and organize multiple cellular activities [2]. Transforming growth factor (TGF-β1) attracts neutrophils, macrophages and fibroblasts in the inflammatory focus [3]. Vascular endothelial growth factor (VEGF) is identified as a major regulator of vasculogenesis and angiogenesis [4], produced by fibroblasts, endothelial cells, platelets, neutrophils and macrophages [5].

Medicinal plants are widely used in folk medicine for the treatment of several injuries and demonstrate efficacy in repair for both normal and burn wounds [6–8]. *Porophyllum ruderale* (Jacq.) Cass. (Asteraceae), commonly known as “arnica paulista”, possesses a variety of biological and pharmacological activities and tinctures or alcoholic leaf extracts of this plant are applied topically in folk medicine as healing or anti-inflammatory agents to treat cuts, wounds and abrasions [9]. Phytochemical studies of the aerial parts of the plant have revealed the presence of quercetin, a glycosidic flavonoid, in addition to tannins saponins, resins and essential oils [10].

The use of low-level laser irradiation (LLLI) to accelerate burn wound healing has been studied and the photobiostimulation has been shown to stimulate reepithelization, collagen synthesis, fibroblast proliferation, angiogenesis and the formation of granulation tissue, and to reduce inflammatory infiltration [11]. However, the ideal parameters of laser photobiostimulation for wound healing are conflicting due to the variety of treatment protocols, animal models and cell cultures used [12]. Nevertheless, laser therapy has been shown to be useful as a coadjuvant, favoring the healing process of different tissues [13].

Considering the beneficial effects of herbal medicines and laser therapy on the wound healing, the objective of the present study was to investigate the cellular responses induced by topical application of extract of *P. ruderale* alone or in combination with 670-nm Indium Gallium Phosphide (InGaP) laser irradiation on the healing of burns produced on the back of rats.

## Methods

### Plant collection and extraction

*P. ruderale* leaves were collected, early in the morning period in the month of in September (2011), in the Medicinal Plant Garden of Hermínio Ometto University Center, UNIARARAS, Araras, São Paulo, Brazil. A voucher specimen was deposited at the herbarium of the Luiz de Queiroz College of Agriculture (Escola Superior de Agricultura Luiz de Queiroz—ESALQ-USP) (n^o^ ESA115686).

After collection, the leaves (100 g) were selected and properly cleaned under running water to remove impurities. The hydroalcoholic extract of *P. ruderale* was prepared by maceration of the leaves in a hydroalcoholic solution of 70 % (v/v solution of ethanol in water) for 7 days at room temperature, followed by vacuum filtration using a qualitative paper filter and evaporation in a rotary evaporator (Fisatom, model 803) at 40 °C. The evaporation period was 1 h. The resulting samples were subjected to lyophilization [14] and the yield of the lyophilized extract was from 10 %, ie, after all proceedings of extraction, the 100 g of leaves yield 10 g of the lyophilized. Obtaining this extract was based on popular culture [15].

### Phytochemical screening method

The qualitative identification of chemical constituents was carried out in the same extract as that used in the wound repair test using chemical methods [14] and thin-layer chromatography [16]. The chemical groups analysed were polyphenolic components, flavonoids, tannins, alkaloids, saponins, fatty acid, triterpenes, volatile oils, coumarins and anthraquinones.

The presence of polyphenol compounds was analyzed with a solution of 1 % ferric chloride. Tannins have been identified using the dried extract dissolved in water, 2 ml of sodium chloride (2 %); filtered and mixed with 5 ml of 1 % gelatin. The presence of flavonoids was determined using aluminum chloride solution 1 % in methanol, concentrated hydrochloric acid, magnesium and potassium hydroxide. Dragendorff reagent was used to evaluate the presence of total alkaloids. Saponins were analyzed based on its ability to produce foam. For detection of triterpenes extract was mixed with 5 ml of chloroform was heated to 80 °C for 30 min and then treated with a small volume of concentrated sulfuric acid. Moreover, the extract was analyzed by thin layer chromatography on silica gel using chloroform: methanol (98:2) and hexane:ethyl acetate (80:20) as eluent. The components were first visualized under UV light and then by spraying the chromatographic plates and each containing different specific solutions, followed by incubation at 100 °C for 5 min.

The method of Chandra and Mejia Gonzalez [17] was used for quantitative analysis of total polyphenols. The hydroalcoholic extract (0.1 ml) was added to 1 ml Folin-Ciocalteau reagent and the mixture was left to stand for 5 min. Next, 2 ml 20 % sodium carbonate was added. After incubation for 10 min at room temperature, absorbance of the mixture was read in a spectrophotometer at 730 nm. The amount of total polyphenols is expressed as catechin equivalents.

For the analysis of flavonoids, the hydroalcoholic extract was incubated with 10 % aluminum chloride, 95 % ethanol, 1 mol/l sodium acetate, and distilled water for 30 min at room temperature. Absorbance was read in a spectrophotometer at 425 nm and samples were analyzed in triplicate. The amount of flavonols and flavones is expressed as quercetin equivalents [18].

### Animals

Seventy-two (*Rattus norvegicus albinus*) male Wistar rats aged approximately 120 days and weighing on average 250 g were obtained from the “Prof. Dr. Luiz Edmundo de Magalhães” Animal Experimentation Center, of Hermínio Ometto University Center, UNIARARAS. The animals were housed in individual polycarbonate cages at a constant temperature (23 ± 2 °C) and humidity (55 %) under a 12-h light/dark cycle, with free access to standard chow and drinking water. All surgical and experimental procedures were approved by the Ethics Committee of Hermínio Ometto University Center, UNIARARAS (Protocol number 092/2011) and were conducted in accordance with the ethical guidelines of the Brazilian College of Animal Experimentation (COBEA) and of Guide for the Care and Use of Laboratory Animals (NIH).

### Experimental design and burn wound creation

The animals were divided randomly into four groups of 18 animals each: group **C**, untreated control; group **L**, treated with 670-nm InGaP laser; group **P**, treated with the hydroalcoholic *P. ruderale* extract; group **PL**, treated with the hydroalcoholic *P. ruderale* extract and 670-nm InGaP laser (extract applied prior to laser therapy).

For experimental wounding, the back of the animals was shaved 48 h before the procedure under general anesthesia induced by the intraperitoneal administration of ketamine (1.0 ml/kg) and xylazine (0.2 mg/kg). Skin burns were produced on the backs of all animals, after the same anesthetic procedure, by applying an aluminum plate measuring 2 cm in diameter adapted to an apparatus that maintained a constant temperature of 120 °C. The plate was pressed on the animal’s skin for 20 s for the creation of a second-degree burn [19]. After induction of burns, the animals received pain killers: sodium dipyrone, one drop in the postoperative, after 12 and 24 h and they were placed in individual cages. Besides the treatments for repair of burns was not carried out any specific care except individualize the animals throughout the experimental procedure. The treatments were started 24 h after injury and were applied daily at the same time for 21 days. The animals were immobilized without sedation for treatment. The control (untreated) animals were subjected to sham treatments to control for effects of immobilization without treatment.

For laser therapy, an InGaP laser (Phisiolux Dual Bioset®, Indústria de Tecnologia Eletrônica Ltda., Rio Claro, São Paulo, Brazil) operating at a wavelength of 670 nm (visible red) was applied in the continuous mode using the following parameters: power output of 30 mW, energy density of 4.93 J/cm^2^ and total energy dose of 0.36 J, with the laser beam covering an area of 0.073 cm^2^. Punctual irradiation was performed by non-contact energy delivery at a distance of ± 2 mm and an angle of 90° in relation to the wound surface. The time of application (12 s) was determined by the specification of the equipment and the laser was applied to four points within the burn area. The apparatus was calibrated by the manufacturer.

The hydroalcoholic extract of *P. ruderale* (1.0 ml/day) was applied with a Pasteur pipette to the borders of the wound.

### Structural and morphometric analyses

Wound samples were collected 7, 14 and 21 days after injury from three animals per group euthanized with an overdose of the anesthetic. An area measuring 25 mm in diameter was delimited in the center of the wound to obtain standardized samples for structural and morphometric analysis.

The tissue fragments were immersed in fixative solution containing 10 % formaldehyde in Millonig buffer, pH 7.4, for 24 h at room temperature. Next, the specimens were washed in buffer and submitted to routine procedures for embedding in Paraplast™ (Histosec®, Merck, Darmstadt, Germany). The blocks were cut into 6-μm longitudinal sections.

Longitudinal sections stained with Toluidine Blue and by the Domini method [20] were used to determine the number of fibroblasts and granulocytes and newly formed vessels in the repair area of the groups studied (n/10^4^ μm^2^). Three samplings were performed from each of the five sections obtained from whole sections of the middle part of each surgical sample. The first 16 sections of the mid-section of the surgical specimen collected from each animal per group were collected. The sections were mounted on eight slides and were chosen randomly for the methods described above. After staining, three samplings of 10^4^ μm^2^ (100 × 100 μm grid) were performed per section obtained in each test. Each sample was photomicrographed and digitized bright-field images were obtained with a Leica DM2000 photomicroscope at the Laboratory of Micromorphology, Hermínio Ometto University Center, UNIARARAS. Masson’s trichrome staining was used to quantify collagen fiber content in the repair area (percentage of total area). The fields were separated using the blue color distribution as a discrimination parameter. The intensity of blue represents the collagen density. The color band was adjusted by trial and error until representative areas of collagen had been separated in the image**.** The same parameter was then used to identify collagen fibers in all digitized fields. Next, the area occupied was calculated for each field [21].

Samples measuring 10^4^ μm^2^ (100 × 100 μm grid) obtained from the area of the wound healing on days 7, 14 and 21 of treatment were examined using the Leica Image Measure™ visual grid for morphometric analysis of the following parameters: total number of fibroblasts, granulocytes, newly formed blood vessels (n/10^4^ μm^2^), and collagen fiber content (% area). The Sigma Scan Pro 6.0™ program was used to evaluate the deposition of granulation tissue in the repair area. The results were entered into Excel for Windows XP™ spreadsheets and compared by ANOVA and the Tukey post-test (*p* < 0.05) [22].

### Western blotting analysis

For analysis of protein expression by Western blotting, wound samples were collected from three animals per group after euthanasia with an overdose of the anesthetic, on days 7, 14 and 21 of treatment. For protein extraction, the samples obtained were chopped and homogenized in a Polytron homogenizer (PTA 20S model PT 10/35; Brinkmann Instruments, Westbury, NY, USA) operated at maximum speed for 40 s in buffer (10 mM EDTA, 100 mM Trizma base, 10 mM sodium pyrophosphate, 100 mM sodium fluoride, 100 mM sodium orthovanadate, 2 mM PMSF, 0.1 mg/ml aprotinin, and deionized water; Sigma Chemical Co., USA). The extract was centrifuged at 12,000 rpm for 20 min at 4 °C for removal of insoluble material. The supernatant was collected for the measurement of protein concentration in the samples by the Biuret method (Protal colorimetric method, Laborlab, São Paulo, Brazil). Aliquots of the supernatant were treated with Laemmli buffer containing 100 mM DTT (Sigma Chemical Co., St. Louis, MO, USA). Samples containing 50 μg protein were boiled for 5 min and submitted to 10 % (VEGF, 40 kDa) and 12 % (TGF-β1, 25 kDa) SDS-PAGE (Sodium dodecyl sulfate-polyacrylamide gel electrophoresis) in a mini-gel apparatus (Mini-Protean®, Bio-Rad-Richmond, CA, USA). Next, the protein bands were transferred from the gel to a nitrocellulose membrane (Hybond ECL, 0.45 μm) [23]. The membranes were washed in basal solution (1 M Trizma base, 5 M NaCl, Tween 20 a 0,005 %, and deionized water) and incubated in blocking solution (basal solution plus 5 % Molico® skim milk) for 2 h to reduce nonspecific protein binding. After washing with basal solution, the membranes were incubated overnight at 4 °C with specific antibodies (diluted 1:200) Anti-TGF-β1 (TB21, Santa Cruz Biotechnology, USA) and Anti-VEGF (VG-1, Santa Cruz Biotechnology, USA). Next, the membranes were incubated with the secondary goat anti-mouse IgG1:HRP antibody (diluted 1:1000, Santa Cruz Biotechnology, USA) for 2 h at room temperature. The reaction was developed using a chemoluminescence kit (SuperSignal® West Pico Chemiluminescent Substrate 34080, Thermoscientific, Rockford, USA). The membranes were exposed in Syngene G: Box and the bands intensity were evaluated by densitometry using the Scion Image 4.0.3.2 software (Scion Co., USA). The densitometric values of VEGF and TGF signals are expressed relative to proteins stained with Ponceau S, which were taken as 100 %. The results were analysed by ANOVA and the Tukey post-test (p < 0.05) 0.29 using the GraphPad Prism® 3.0 program.

## Results

### Phytochemical screening

The following classes of chemical compounds were identified in the extract: flavonoids, tannins, saponins, fatty acids. Alkaloids, volatile oils and anthranoids were also detected. The total polyphenols content was 2.29 μg catechin equivalents/ml and the flavonols and flavones content was 0.625 μg quercetin equivalents/ml.

### Structural and morphometric analyses of wound repair

The repair process after thermal injury, in both groups, was studied by comparing the inflammatory processes (leukocytosis, hemorrhage and exudate), proliferative (fibroblast hyperplasia, angiogenesis and re-epithelialization) besides the tissue reorganization in the samples obtained on the 7, 14 and 21 days of treatment.

**Fig. 1:**
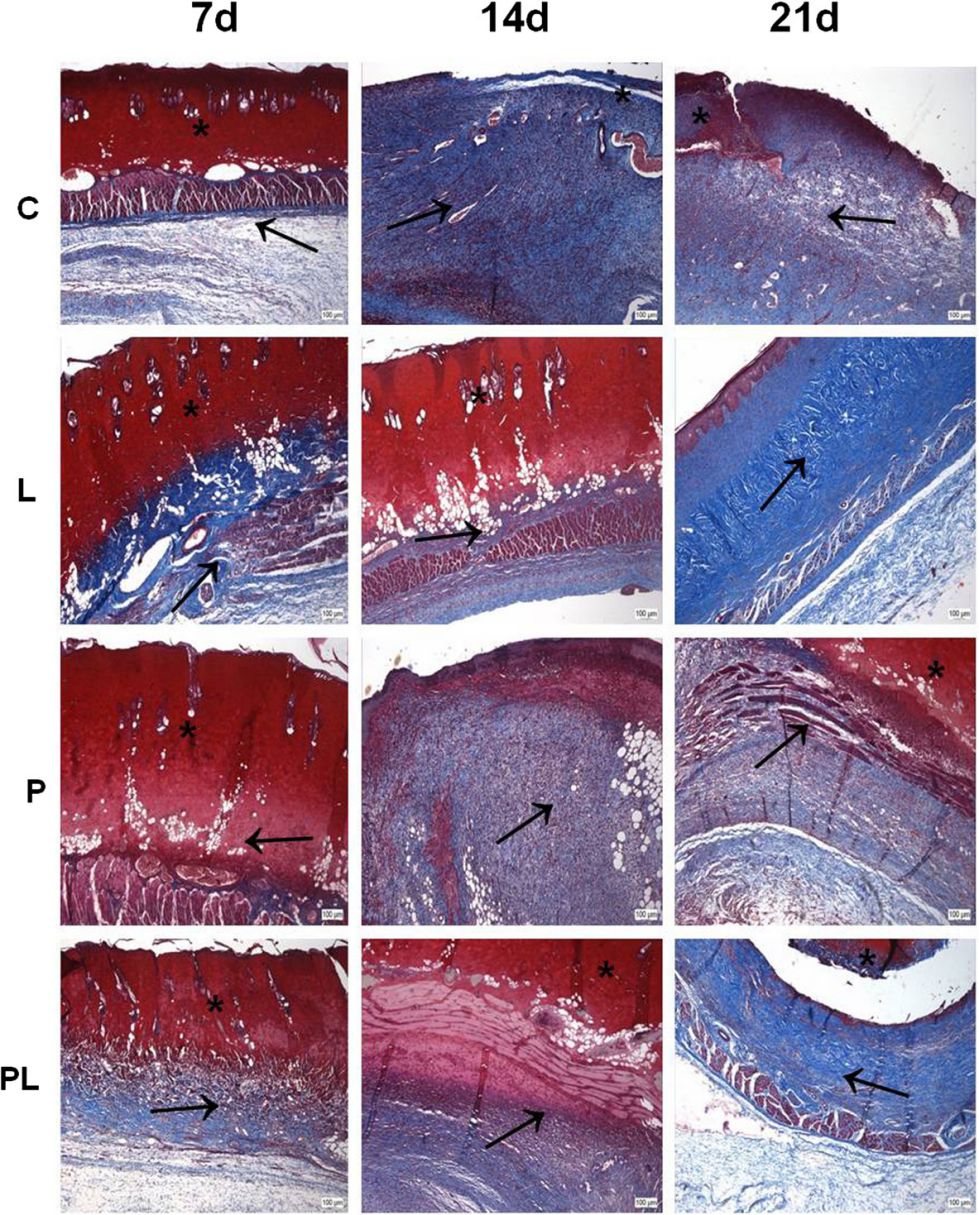
Cross-sections of second-degree burns in rats in different treatment groups. C: untreated control; L: treated with 670-nm InGaP laser; P: treated with the *Porophyllum ruderale* extract PL: treated with the *P. ruderale* extract and 670-nm InGaP laser. Samples were collected from each group 7, 14 and 21 days after injury. The sections were stained with Masson’s trichrome. (*) Wound area; (→) collagen fibers. Bar = 100 μm

Qualitative analyses of samples collected from the lesion area of the different groups showed newly formed granulation tissue on day 14 and at day 21. In this period, the structural characteristics of the repair tissue indicate that the reorganization phase is already established in this period. There were no hemorrhages or edema on the different experimental periods. Deposition and reorganization of collagen fibers in the repair area showed increasing compaction and reorganization levels. Proliferation of new epithelium can be observed starting from the 7th day. The new layers of cells originating from the lesion edges gradually occupied the entire surface of the lesion to re-cover it completely in 21 days (Fig. 1).

Quantitative analysis of the data of all experimental groups showed an increase in the number of fibroblasts during the period studied with significant difference compared to the groups treated with laser (**L**) and this combined with *Porophyllum ruderale* (**PL**) (Table 1).

**Table 1:** Cellular parameters evaluated in the wound healing process of second-degree burns in rats in different treatment groups

Cellular parameters/Experimental groups		C	L	P	PL
Number of fibroblasts (n/10^4^ μm^2^)	Experimental periods				
	7d	15.3 ± 3.1	22.3 ± 2.1*	16.1 ± 3.2	21.9 ± 3.4* ^p=0.037^
	14d	24.2 ± 4.4	31.6 ± 3.1*	23,3 ± 2.3	30.7 ± 3.2* ^p=0.043^
	21d	28.5 ± 4.1	37.2 ± 3.3*	29.1 ± 4.2	38.2 ± 3.4* ^p=0.040^
Number of granulocytes (n/10^4^ μm^2^)	7d	26.1 ± 2.4 *^p=0.043^	21.5 ± 2.4	21.3 ± 2.3	22.1 ± 2.3
	14d	18.7 ± 2.2	19.5 ± 3.8	17.7 ± 3.1	18.5 ± 3
	21d	9.6 ± 3.1	7.2 ± 2.5	8.1 ± 2.2	8.2 ± 2.9

Samples were collected from each group 7, 14 and 21 days after injury. Values are the mean and standard deviation of each group and were compared by ANOVA with Tukey’s post-test

*C* untreated control, *L* treated with 670-nm InGaP laser, *P* treated with the *Porophyllum ruderale* extract, *PL* treated with the *P. ruderale* extract and 670-nm InGaP laser

Asterisk (*) indicates significant differences (*p* < 0.05)

A significantly larger number of granulocytes were observed in untreated wounds at the beginning of wound healing (day 7). The number of these cells decreased gradually during the experimental period in all groups (Table 1).

Significantly higher collagen fiber content was observed in group **L** on day 21 and in group **PL** on days 14 and 21 when compared to the other groups (Table 2).

**Table 2:** Collagen content (% of area) evaluated in the wound healing process of burns second degree in rats in different treatment groups

Experimental groups/periods	C	L	P	PL
7d	20.5 ± 8.2	28.6 ± 7.2	22.6 ± 4.7	29.4 ± 5.6
14d	41.3 ± 8.1	39.6 ± 8.2	37.6 ± 5.7	56.9 ± 6.8* ^p=0.048^
21d	53.4 ± 9.5	88.6 ± 10.4* ^p =0.046^	48.8 ± 7.8	71.5 ± 7.2*

Samples were collected from each group 7, 14 and 21 days after injury. Values are the mean and standard deviation of each group and were compared by ANOVA with Tukey’s post-test

*C* untreated control, *L* treated with 670-nm InGaP laser, *P* treated with the *P. ruderale* extract, *PL* treated with the *P. ruderale* extract and 670-nm InGaP laser

Asterisk (*) indicates significant differences (*p* < 0.05)

The total number of newly formed vessels in the tissue repair area was significantly higher in samples of group **L** in all experimental periods when the data of all groups were compared (Table 3).

**Table 3:** Number of new vessels (n/10^4^ μm^2^) evaluated in the wound healing process of burns second degree in rats in different treatment groups

Experimental groups/periods	C	L	P	PL
7d	1.1 ± 0.2	1.8 ± 0.3* ^p=0.044^	1.0 ± 0.4	1.2 ± 0.2
14d	1.7 ± 0.4	2.7 ± 0.3* ^p=0.042^	1.4 ± 0.7	2.2 ± 0.1
21d	2.2 ± 0.4	3.1 ± 0.4* ^p=0.045^	2.2 ± 0.3	2.0 ± 0.4

Samples were collected from each group 7, 14 and 21 days after injury. Values are the mean and standard deviation of each group and were compared by ANOVA with Tukey’s post-test

*C* untreated control, *L* treated with 670-nm InGaP laser, *P* treated with the *P. ruderale* extract, *PL* treated with the *P. ruderale* extract and 670-nm InGaP laser

Asterisk (*) indicates significant differences (*p* < 0.05)

### Western blotting analysis

**Fig. 2:**
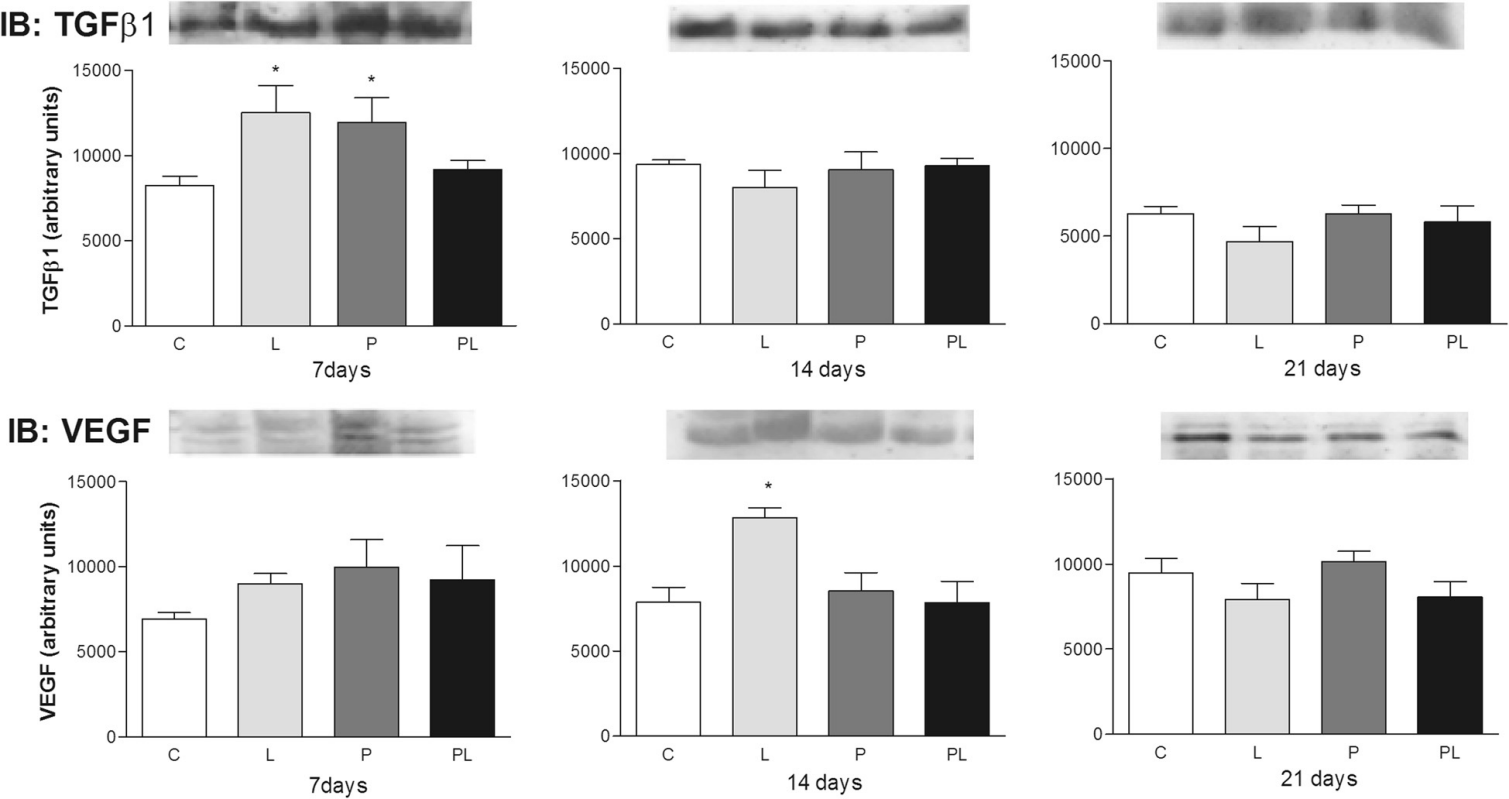
Immunoblot analysis of the expression of TGF-β1 and VEGF after 7, 14 and 21 days of the experiment in second-degree burns in rats in different treatment groups. C: untreated control; L: treated with 670-nm InGaP laser; P: treated with the *Porophyllum ruderale* extract PL: treated with the *P. ruderale* extract and 670-nm InGaP laser. Typical blots are shown above average densitometry results. Values are the mean and standard deviation of each group and were compared by ANOVA with Tukey’s post-test (* *p* < 0.05)

The expression of TGF-β1 and VEGF (Fig. 2) proteins evaluated by Western blotting showed some differences demonstrated by densitometry. A significant increase in the expression of TGF-β1 was observed at the beginning of the repair process (day 7) in wounds submitted to laser irradiation and application of the *P. ruderale* extract when compared to the other groups. On days 14 and 21, the expression of this growth factor was reduced at the samples of lesions in all experimental groups. Particularly on samples irradiated with laser it is possible to observe a decrease in the expression of TGFβ-1, as compared to other groups on day 21 (Fig. 2).

Differences in the expression of VEGF were observed throughout the experimental period in the treated groups. There was a significant increase in VEGF expression in the laser-irradiated group (L) on day 14 when compared to control, and was reduced on day 21. Treatment with extract of *P. ruderale* on day 7 increased expression of this protein compared to control, but decreased in day 14 (Fig. 2).

## Discussion

Experimental studies have shown that some phytotherapeutic agents are effective in accelerating the healing of burns [24, 25]. *P. ruderale* is used in the folk medicine for cicatrization and its anti-inflammatory properties and cicatrizing activity [26]. Similarly, many experimental studies have found beneficial results with the use of low-level laser irradiation on burn wound healing [11]. The aim of the combination of this medicinal plant with laser exposure was to evaluate possible positive synergic effect among these therapies on the healing of burns in rats as observed by Mendonça et al. [27] in the treatment of wound in sheep in which the authors obtained a reduction in repair time of these lesions.

In this study, the number of fibroblasts and collagen fibers increased in the burns treated with 670-nm InGaP laser alone and in those treated with both laser and *P. ruderale* extract. There was no increase in either fibroblasts or collagen fibers in the wounds that were either untreated or treated with the extract alone. This suggests that the laser treatment promoted a more effective response. Similarly, other studies have observed that low-intensity laser treatment of wounds in rats also promotes increased collagen production as a result of enhancement of fibroblast proliferation [28]. During reepithelialization the cell proliferation is an essential event so proliferating fibroblasts at the wound site ensure an adequate supply of cells that will migrate and cover the wound surface [29].

Quantitative analysis revealed a significant increase in the number of newly formed vessels in wound samples treated only with the 670-nm InGaP laser. These results are in accordance with the literature that considers the laser as an angiogenesis stimulator [30]. Neves et al. [31] studied the effect of laser irradiation 670-nm InGaP on excisional wounds in Wistar rats and observed that this approach also promoted an increase in angiogenesis at the injury site. Similarly, Sousa et al. [32] observed significant angiogenic effects promoted by low-level laser therapy in skin burns and excisional wounds on the back of Wistar rats, respectively.

The combination of laser with *P. ruderale* did not promote the same answers that L and P groups. In PL group was observed fibroblast proliferation and anti-inflammatory action. However, a synergistic effect was not observed between these two agents as expected when the experimental design was conceived. A probable explanation for this fact may be related to the permanence of the green pigment of leaves in *P. ruderale* extract, even after lyophilization. This may have partially compromised the laser performance in the tissue, once when the laser was applied alone it was possible to observe its effectiveness in increasing the content of collagen fibers and the number of newly formed vessels in the injured area. According to the considerations of Melo et al. [33] despite experimental difficulties in evaluating the light transport in biological tissues, it is important to know the absorption and light scattering coefficient in these, which are fundamental quantities that enable the understanding of the various effects of light-tissue interaction. These authors have experimentally used different types of lasers to evaluate the exponential attenuation of the laser light during its propagation in different rat tissues. High penetration of 630 nm laser red light in the abdominal fat layer of rat was observed and it was found that this was due to the absence of pigments in this tissue which facilitated its penetration [33]. Differently, Prates et al. [34] investigate the ability of malachite green combined with a low-power red laser (GaAlAs 660 nm) to kill *Actinobacillus actinomycetemcomitans* and showed that an important periodontal bacterium can be photoinactivated by red light and malachite green. Malachite green (MG) shows strong absorption at the red end of the visible spectrum [35].

Different studies demonstrated that low-intensity laser, used experimentally to repair wounds, promotes decrease in the number of inflammatory cells [36, 37] which agrees with our results found through morphometric analyzes of the samples from the lesions treated with laser irradiation. Lesions samples treated with the extract of *P. ruderale* (“arnica paulista”) results were similar to other samples of the wounds from groups submitted to treatment, having reduced significantly the number of granulocytes cells when compared to untreated wounds during of the repair process. This result confirms other studies that have been made with other species of the Asteraceae family, for example, *Solidago microglossa* (brazilian arnica) [38] and *Arnica montana* [39] showed anti-inflammatory effects on skin wounds in Wistar rats. Probably due to the presence of flavonoids in the composition of arnicas, indicating the anti-inflammatory properties of this plant. Flavonoids are known for their antioxidant and anti-inflammatory properties [40] and Park et al. [41] also described the high therapeutic potential of these compounds for thermal skin injuries in mice. The phytochemical analysis in our study showed a large amount of phenolic compounds in the leaves of *P. ruderale*, and Conde-Hernandez and Guerrero-Beltrán [10] considered that these coumpounds may be responsible for the antioxidant activity of this plant.

Despite the large number of reports on the *in vitro* and *in vivo* actions of TGF-β1, there is still controversy regarding the endogenous role of this growth factor which seems to be involved in the onset of the inflammatory phase during wound healing [42]. Neves et al. [31] observed an increase in TGF-β1 expression at the beginning of the healing process (day 6) and a reduction on day 10 of treatment with 670-nm InGaP laser. This confirms our results that showed expression of this cytokine highlighted in the early phases of the healing of burn injuries in the samples treated with laser and also with the extract of *P. ruderale*. The Asteraceae family (arnicas) favors wound healing because promote an anti-inflammatory response due to the presence of flavonoids in their constituents [43] and probably these compounds, as well as laser irradiation, favored the release of this protein at this stage of the healing process.

TGF-β1 has potent regulatory activity of inflammatory processes since it exerts a chemoattractant role for neutrophils and macrophages, which are important in the initial processes of tissue repair [3, 44]. Wang et al. [45] investigated the healing of skin wounds in mice and suggested that the excessive and prolonged presence of TGF-β1 at the site of injury does not benefit wound healing. This is partially due to the proinflammatory effect of this protein [45]. The decrease in TGF-β1 expression from day 21 lesions treated with 670-nm InGaP laser (**L**) may indicate that this treatment benefited the repair process by reducing the time of the inflammatory phase contributing to faster healing.

Angiogenesis is modulated by hypoxia, nitric oxide and growth factors such as VEGF [46] which is produced by different types of cells that participate in wound healing. Differences in the expression of VEGF were observed throughout the experimental period in the treated groups. There was a significant increase in VEGF expression in the laser-irradiated group (L) on day 14 when compared to control, and was reduced on day 21. Treatment with extract of *P. ruderale* maintained the expression of this protein throughout the experimental period similarly to the control (Fig. 2).

In our study we found that the expression of VEGF has been modified in the irradiated samples with a significant increase at day 14 of treatment, indicating that in this period the laser therapy promoted angiogenesis, since this growth factor is implicated in this process during skin development [47]. Neves et al. [31] compared the effects of microcurrent stimulation and LLLT on experimental wound healing in healthy and diabetic Wistar rats. The authors observed an increase in VEGF expression in all diabetic groups on day 6 when compared to the respective groups of healthy animals. This finding might be related to the tissue hypoxia commonly seen in the skin of diabetic individuals as a consequence of impaired microcirculation since tissue hypoxia significantly increases the expression of VEGF and this may be responsible for improved tissue repair. Renno et al. [48] irradiated second-degree burns in rats with a low-level laser (660 nm) and observed significant expression of VEGF on day 14. The authors suggested that this treatment was beneficial since it reduced the necrotic area in the wound through angiogenesis.

## Conclusions

The results of this study indicate that laser irradiation 670-nm InGaP promoted beneficial responses in the repair process increasing collagen deposition and angiogenesis when was used separately. *Porophyllum ruderale* was effective in decreasing the granulocytes during the repair process indicating a possible anti-inflammatory action of this native flora, widely used in folk medicine, but little studied experimentally.
